# Carbohydrates and ginsenosides in shenmai injection jointly improve hematopoietic function during chemotherapy-induced myelosuppression in mice

**DOI:** 10.1186/s13020-022-00678-5

**Published:** 2022-11-04

**Authors:** Shiyu Zhang, Yinxiao Mi, Tingting Ye, Xiaoyan Lu, Li Liu, Jing Qian, Xiaohui Fan

**Affiliations:** 1 Pharmaceutical Informatics Institute, College of Pharmaceutical Sciences, Zhejiang University, Hangzhou, 310058 China; 2 Chiatai Qingchunbao Pharmaceutical Co. Ltd, Hangzhou, 310030 China; 3 Jinhua Institute of Zhejiang University, Jinhua, 321016 China; 4 Innovation Institute for Artificial Intelligence in Medicine of Zhejiang University, Hangzhou, 310058 China; 5 State Key Laboratory of Component-Based Chinese Medicine, Tianjin University of Traditional Chinese Medicine, Tianjin, 301617 China

**Keywords:** Bone marrow stromal cells, Carbohydrates, Myelosuppression, RNA sequencing, Shenmai injection, Traditional Chinese medicine

## Abstract

**Background:**

Shenmai injection (SMI), a traditional Chinese medicine (TCM) injection prepared from *Red ginseng* and *Ophiopogon japonicus*, is widely used in clinics to treat chemotherapy-induced myelosuppression. Similar to other TCM injections, SMI contains a high amount of carbohydrates (fructose, sucrose, and maltose) in addition to the bioactive substances, specifically ginsenosides (Rg1, Re, and Rb1). To date, the role of these carbohydrates in the hematopoietic function of SMI remains unclear.

**Purpose:**

We aimed to investigate the hematopoietic effects and potential mechanisms of SMI and its components, focusing on the carbohydrates present in SMI.

**Experimental design/methods:**

First, we evaluated the hematopoietic effect of SMI on 5-fluorouracil (5-FU)-induced myelotoxicity in a tumor-bearing mouse model. Then we prepared mixtures of ginsenosides and carbohydrates according to their proportions in SMI and evaluated their hematopoietic function in mice with 5-FU-induced myelosuppression. Finally, hematopoiesis-related molecular networks were built based on RNA sequencing (RNA-seq) of the bone marrow stromal cells (BMSCs), and the potential mechanisms of carbohydrates and ginsenosides were evaluated.

**Results:**

SMI attenuated 5-FU-induced myelotoxicity in tumor-bearing mice. Both ginsenosides and carbohydrates increased the bone marrow nucleated cell (BMNC) count and improved the bone marrow morphology in myelosuppressive mice; they promoted the proliferation of BMSCs derived from those myelosuppressive mice. Bioinformatics analyses revealed ECM-receptor interaction, Hippo signaling, and Wnt signaling are common pathways regulated by both ginsenosides and carbohydrates; *Gstt1, Gstp2, Gsta4* and *Oplah* in Glutathione metabolism pathway and *Cd19, Cd79a,* and *Cd79b* in B cell receptor pathway are uniquely regulated genes related to carbohydrates but not ginsenosides.

**Conclusions:**

Carbohydrates may collaborate with ginsenosides and contribute to the hematopoietic function of SMI. Carbohydrates could be considered as a bioactive component in this TCM injection.

**Graphical Abstract:**

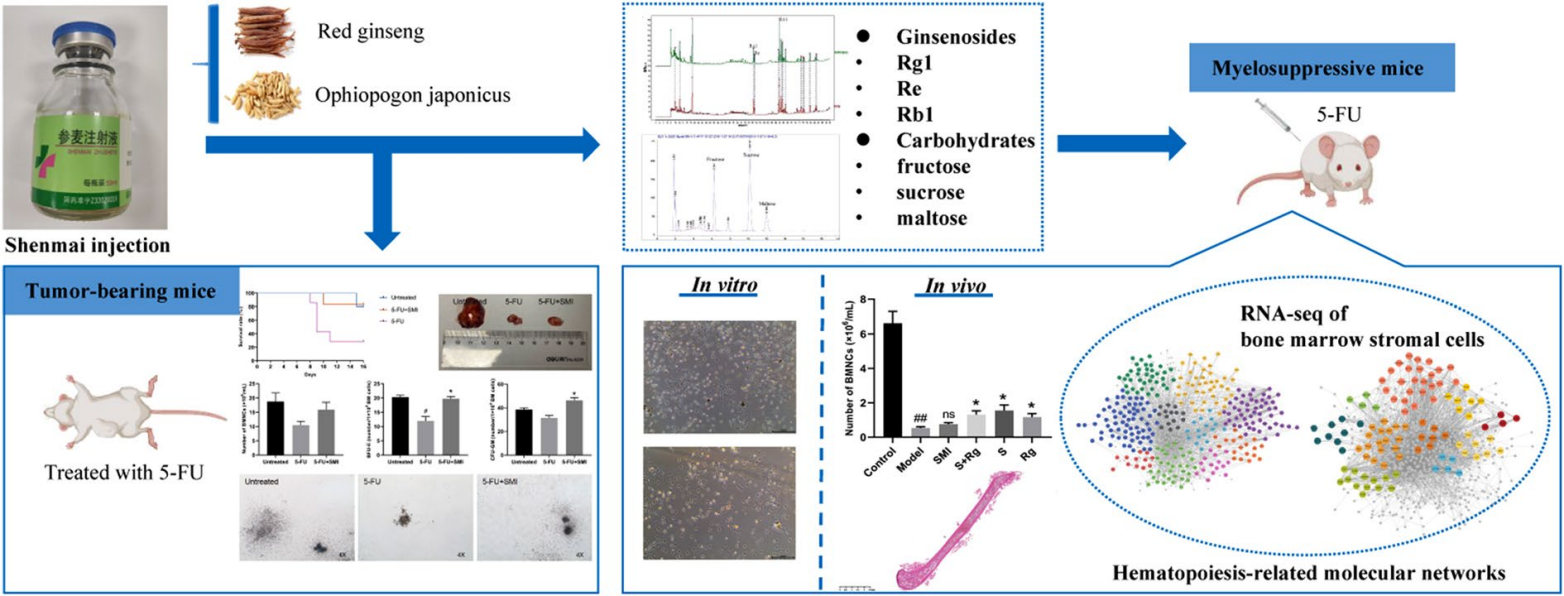

**Supplementary Information:**

The online version contains supplementary material available at 10.1186/s13020-022-00678-5.

## Highlights

- Shenmai injection attenuates 5-FU-induced myelotoxicity in tumor-bearing mice.
- Both carbohydrates and ginsenosides in Shenmai injection promote hematopoiesis.
- Carbohydrates may regulate Glutathione metabolism and B cell receptor pathway.

**Supplementary Information:**

The online version contains supplementary material available at 10.1186/s13020-022-00678-5.

## Introduction

Myelotoxicity is a major side effect of chemotherapeutic drugs such as 5-fluorouracil (5-FU). This chemotherapeutic agent targets DNA in proliferating cells and, while killing cancer cells, also acts on other highly proliferative tissues such as the bone marrow, resulting in a high incidence of myelotoxicity leading to chemotherapy delays or discontinuations [1]. Methods or drugs that reduce the side effects of chemotherapy are under extensively exploration.

Chinese herbal medicines are widely used in clinical cancer therapy to alleviate chemotherapy-related side effects [2]. Shenmai injection (SMI) is a traditional Chinese medicine (TCM) injection derived from Shengmai San originally recorded in Yixue Qiyuan. SMI is prepared from two herb medical materials: *Red ginseng* (Hongshen, the dried roots and rhizomes of *Panax ginseng* C.A.Mey.) and *Ophiopogon japonicus* (Maidong, the dried roots of *Ophiopogon japonicus* (Thunb.) Ker-Gawl.); it has been widely used to treat diseases such as viral myocarditis, shock, and granulocytopenia [3]. While used with chemotherapeutic drugs, SMI benefits the cancer patients from both enhanced anti-tumor activity and relieved myelotoxicity [4, 5]. Nevertheless, as a common challenge of TCM, the pharmacological activities and the bioactive components of SMI are poorly understood.

SMI contains various chemical constitutions including ginsenosides, ophiopogonins and flavones etc. [6]. Among them, ginsenosides Rg1, Re, and Rb1 are recognized as bioactive compounds and listed as quality indicators for SMI in *Chinese Pharmacopoeia 2020*. The protective role of ginsenosides Rg1, Re, and Rb1 in myelosuppression has been intensively investigated: Rg1 improves the hematopoietic function of the bone marrow; therefore, it reduces myelosuppression caused by cyclophosphamide [7]. Re and its secondary metabolite, Rk3, can alleviate cyclophosphamide-induced myelosuppression by regulating cytokine levels and the Bcl-2/Bax balance [8]. Although a direct effect of Rb1 on myelosuppression has not been reported, the main metabolite, ginsenoside compound K (CK), generated by the action of Rb1 in the intestine, can attenuate cyclophosphamide-induced myelosuppression [9]. In addition, ginsenosides Rg1, Re and Rb1 have been identified as potential effective components in an herb pair preparation of ginseng and *Ligustrum lucidum* Ait that treat myelosuppression [10]. Of note, SMI contains a large amount of carbohydrates, including glucose, sucrose, fructose, and maltose [11]. In addition to providing energy in the form of glucose, till now, the role of carbohydrates in the hematopoietic function of SMI remains unclear.

In the current study, we investigated the hematopoietic effects and potential mechanisms of SMI and its compounds, focusing on the carbohydrates present in SMI. First, we evaluated the ability of SMI to attenuate 5-FU-induced myelotoxicity in tumor-bearing mice. Then we prepared mixtures of ginsenosides and carbohydrates according to their proportions in SMI and evaluated their hematopoietic function. Finally, hematopoiesis-related molecular networks were built based on RNA sequencing (RNA-seq) of the bone marrow stromal cells (BMSCs), and the potential mechanisms of carbohydrates and ginsenosides and their contribution to SMI were evaluated.

## Materials and methods

### Preparation of SMI, mixtures of ginsenosides and carbohydrates

SMI (Med-Drug Permit No. Z33020019) was obtained from Chiatai Qingchunbao Pharmaceutical Company (Hangzhou, China). SMI was prepared as described in the national standards approved by the National Medical Products Administration of China (Approval No. WS3–B-3428–98-2010). The product is a transparent liquid contains 0.2 g crude medicines (0.1 g of Red ginseng and 0.1 g of Ophiopogon japonicus) per 1 mL. The HPLC fingerprint results for SMI (Lot No. 1907018, 1907229, and 1907277) and the determination result of carbohydrates in SMI (Lot No. 1907018) are shown in Additional file 1: Fig. S1 and Additional file 2: Fig. S2, respectively.

At clinics, SMI is administrated to patients intravenously at the dosage of 100 mL/d. The relevant dose for mice is 24.6 mL/kg body weight (bw) administrated intraperitoneally (i.p.) [12]. To avoid the impact of excessive dosing volume in mice, we concentrated the solution of SMI by resuspending the lyophilized powder prepared from 24.6 mL SMI (Lot No. 1907018) with 10 mL normal saline. In this end, 10 mL/kg of concentrated SMI was determined as the relevant dosage for i.p. injection to mice.

Respect for the facts that ginsenosides Rg1, Re, and Rb1 being the major bioactive compounds and that sucrose, fructose, and maltose constituted most carbohydrates in SMI, we prepared mixtures of ginsenosides and carbohydrates of Rg1, Re, Rb1, fructose, sucrose, and maltose as representative constitutions for SMI. The mixtures were prepared according to their proportions in SMI (Lot No. 1907018, Additional file 4: Table. S1). For administration to the mice, all the solutions were prepared with normal saline at the corrected dosages of 10 mL/kg. For the long-term cell culture experiment, all the solutions were applied to the culture medium at a ratio of 1:100 (v/v).

### Mice and reagents

Balb/c mice (8–10-week-old, 20–22 g) were purchased from Charles River Laboratory Animal Technology Company (Jiaxing, China; License No. SCXK (Zhe) 2019–0001) and housed at the Laboratory Animal Center of Zhejiang University (License No. SYXK (Zhe) 2018–0016) with free access to food and water under standard conditions (22 ± 2 °C and 12 h light/dark cycle). All animal experiments were performed according to the guidelines for care and use of laboratory animals of the Laboratory Animal Center of Zhejiang University, and approved by the Laboratory Animal Welfare Ethics Committee of Zhejiang University.

The chemicals of Re (CAS NO. 52286–59-6, purity ≥ 98.0%), Rb1 (CAS NO. 41753–43-9, purity ≥ 98.0%), sucrose (CAS NO. 57–50-1, purity ≥ 98.7%), D-(-)-fructose (CAS NO. 57–48-7, purity ≥ 98.0%), and maltose (CAS NO. 6363–53-7, purity ≥ 94.4%) were purchased from the Standard Technology Company (Shanghai, China). Rg1 (CAS NO. 22427–39-0, purity ≥ 98.0%) was from the Winherb Medical Science Company (Shanghai, China). Normal saline was purchased from the Kelun Pharmaceutical Company (Anyang, China). 5-FU (CAS NO. 51–21-8, purity ≥ 99.0%) was purchased from the Aladdin Biochemical Technology Company (Shanghai, China). Mouse methylcellulose medium (MethoCult medium) used in colony-forming unit (CFU) assays was purchased from Stemcell Technologies (Vancouver, Canada). The cell culture reagents were purchased from Gibco Life Technologies (Grand Island, NY, USA). Anti-GSTT1 polyclonal antibody (Cat NO. GB113777) for immunohistochemical staining was provided by the Servicebio Company (Wuhan, China).

### Experimental design

A tumor-bearing mouse model was established by subcutaneous injection of CT-26 cells (5 × 10^6^ cells per mouse). After tumors grew to 80–100 mm^3^, mice were randomly divided into three groups: Untreated (n = 5), 5-FU (n = 7), and 5-FU + SMI (n = 6). In the 5-FU group, mice were injected with 40 mg/kg 5-FU every other day from Day 0 to Day 15. In the 5-FU + SMI group, mice were injected with SMI once per day from Day 0 to Day 15 in addition to the 5-FU injection. In the Untreated group, mice were injected with an equal volume of normal saline once per day from Day 0 to Day 15. Mice survival was recorded. On Day 16, the mice were sacrificed. The tumors were isolated and photographed. Bone marrow nucleated cells (BMNCs) were collected for cell count and CFU assay.

Additionally, we established an acute myelosuppressive mouse model using a single injection of 5-FU. The dosage was 180 mg/kg or 200 mg/kg depending on the body weight of the mice. In the first experiment, mice were randomly divided into six groups (n = 3 per group): Control, Model, SMI, S + Rg, S, and Rg. In the Model group, mice were injected with 5-FU on Day 0, and injected with normal saline once per day from Day 0 to Day 6. In the SMI, S + Rg, S, and Rg groups, after 5-FU administration, mice were injected with SMI (SMI), combination of ginsenosides and carbohydrates (S + Rg), carbohydrates (S), and ginsenosides (Rg), respectively, once per day from Day 0 to Day 6. In the Control group, mice were injected with an equal volume of normal saline once per day from Day 0 to Day 6. On Day 7, the mice were sacrificed. The femurs of mice were collected for BMNC count and histopathological analyses. In the next experiment, the mice were randomly divided into six groups (n = 6–9 per group): Control, Model, SMI, S + Rg, S, and Rg. In the Model group, mice were injected with 5-FU on Day 0, and injected with normal saline once per day from Day 0 to Day 9. In the SMI, S + Rg, S, and Rg groups, after 5-FU administration, mice were treated as mentioned above, from Day 0 to Day 9. In the Control group, mice were injected with equal normal saline once per day from Day 0 to Day 9. On Day 10, the mice were sacrificed. BMNCs were collected for BMNC count and BMSCs were harvested for RNA-seq.

### BMNC collection and count

The femurs and tibias of the mice were separated under sterile conditions. Both epiphyses including epiphyseal plates were clipped off. Cells were flushed from the bone marrow cavity with the complete medium (RPMI medium 1640 + 10% fetal bovine serum + 1% penicillin–streptomycin), and single cell suspensions were obtained with 100-µm cell strainers. After lysis of erythrocytes, the BMNC count was determined using the automated cell counter (Thermo Fisher Scientific, Waltham, MA, USA) and trypan blue staining.

### CFU assay

BMNCs were used for hematopoietic progenitor cell colony formation experiments according to CFU assay instructions. The BMNCs from each mouse were resuspended at 2 × 10^5^ cells/mL; 0.3 mL of cell suspension was added to 3 mL of methylcellulose medium, and after vortex mixing, 1.1 mL duplicates of the mixture were inoculated into two 35-mm dishes and cultured at 37 °C, 5% CO_2_ environment. On the 9th day of culture, the colony-forming unit granulocyte-monocytes (CFU-GM) and burst-forming unit-erythroid (BFU-E) cell counts were observed using an inverted microscope and photographed to record colony morphology.

### Histopathological analysis of bone marrow

Mouse unilateral femurs were harvested and sent to Haoke Biotechnology (Hangzhou, China) for hematoxylin–eosin (HE) and Masson staining. The immunohistochemical staining of bone marrow for GSTT1 was performed by the Servicebio Company (Wuhan, China). After staining, the sections were placed in the KF-PRO-005 automatic digital slide scanning system (KFBIO, Ningbo, China) for panoramic scanning, and K-Viewer or CaseViewer was used for image acquisition and analysis.

### Long-term bone marrow culture

In a separate experiment, mice were injected once with 5-FU or equal saline and sacrificed 2 days later. Bone marrow cells were extracted for long-term bone marrow culture. In brief, cells were seeded into 6-well plates at a density of 3.5 × 10^6^ cells per well. After adhesion, cells were treated with SMI, S + Rg, S, and Rg, respectively, and cultured at 37 °C, 5% CO_2_ environment. Half of the medium was changed every week. Cell growth was observed under an inverted microscope, and status was recorded by photographs after continuous culture and observation for 4 weeks.

### RNA-seq and data analysis of BMSCs

BMNCs were seeded in 6-well plates for 24 h; then, the adherent cells were collected to obtain stromal cells. The stromal cells of the Control, Model, S + Rg, S, and Rg groups (n = 2 each) were sent to Beijing Genomic Institution (BGI, Shenzhen, China) for RNA-seq. The original sequencing data were uploaded to the public database of the NCBI BioProject with project ID PRJNA828339 [13]. The Deseq2 algorithm was used to screen differentially expressed genes (DEGs) between Model vs Control and each treatment group vs Model, and fold change and adjusted *p*-value were calculated for each gene between groups. DEGs between groups were selected based on the criteria of absolute fold change ≥ 2 and an adjusted *p*-value ≤ 0.001. KEGG pathway enrichment and GO analyses (cutoff *p* = 0.01) were performed using Metascape [14], and hematopoiesis-related molecular networks were constructed using STRING [15] and visualized using Cytoscape 3.8.2.

### Statistical analysis

The experimental data were processed and plotted using GraphPad Prism software (version 8.0). Statistical analysis of survival was performed using Gehan-Breslow-Wilcoxon test, and other statistical analyses were performed using one-way analysis of variance (ANOVA) and Student’s *t*-test. The data are expressed as the mean ± standard error of the mean (SEM), and statistical significance was set at *p* < 0.05.

## Results

### SMI attenuated 5-FU-induced myelotoxicity in tumor-bearing mice

**Fig. 1:**
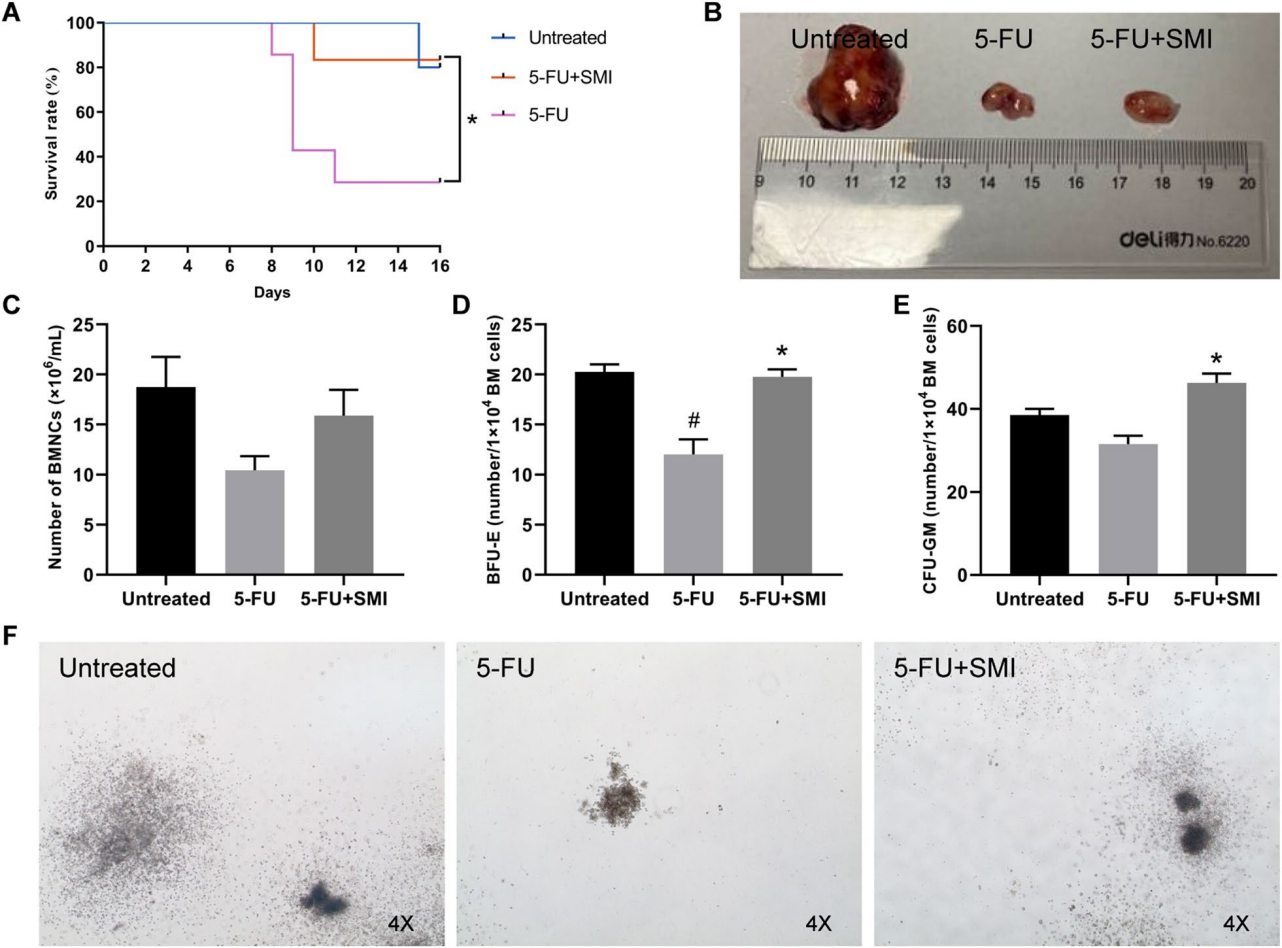
Effects of SMI on 5-FU-induced myelotoxicity. **A** Survival curves for the three groups of mice. 5-FU and SMI treatment started on Day 0. **B** Representative photograph of the largest tumors of the three groups. **C** BMNC count in each group. Cells were isolated on Day 16. **D** BFU-E cell count in each group. **E** CFU-GM cell count in each group. *BFU-E* burst-forming unit-erythroid, *CFU-GM* colony-forming units-granulocyte-monocyte. **F** Representative images of colony formation in each group. The results are expressed as the means ± SEM. ^#^*p* < 0.05, compared with the Untreated group; ^*^p < 0.05, compared with the 5-FU group

Firstly, we tried to verify that SMI could be administrated as a supportive reagent for chemotherapy. In this regard, tumor-bearing mice accepting 5-FU were used to mimic cancer patients undergoing chemotherapy. We found that SMI improved survival of tumor-bearing mice treated with 5-FU (*p* < 0.05) (Fig. 1A) but had no obvious impact on the tumor size (Fig. 1B). In addition, SMI increased BMNC count (Fig. 1C) as well as the colony counts of BFU-E and CFU-GM in mice treated with 5-FU (*p* < 0.05) (Fig. 1D–F). The results indicated that SMI attenuated 5-FU-induced myelotoxicity without affecting the anti-tumor effect of 5-FU.

### Carbohydrates promoted the hematopoietic function in myelosuppressive mice

**Fig. 2:**
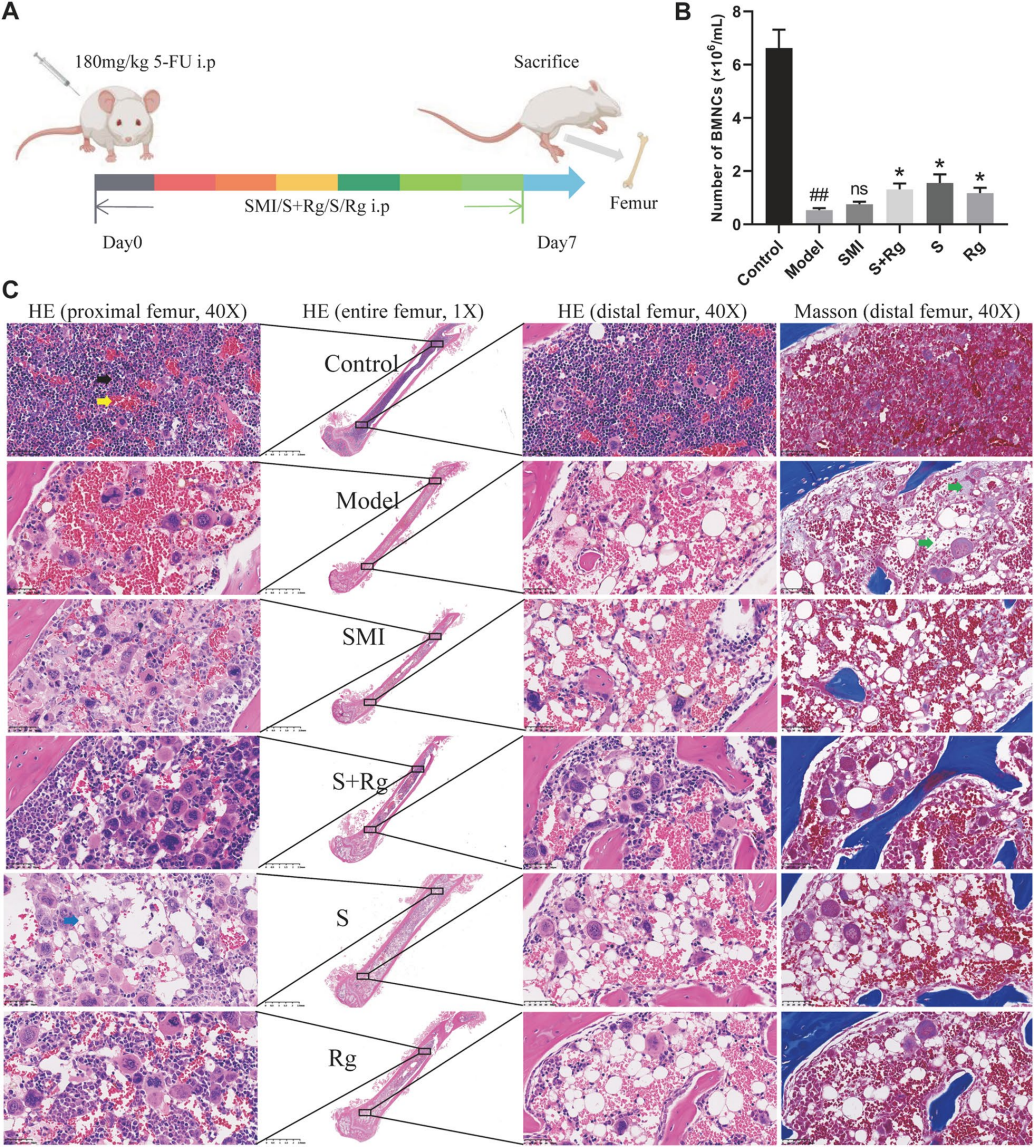
Effects of SMI, ginsenosides, and carbohydrates on hematopoiesis in mice with 5-FU-induced acute myelosuppression. **A** Experimental design. Specific administration is described in “Materials and Methods”. **B** BMNC count in each group. Cells were isolated on Day 7 (n = 3 for each group). The results are expressed as the means ± SEM. ^##^*p* < 0.01, compared with Control group; ^*^p < 0.05, compared with Model group; ns, no significant difference compared with Model group. **C** Representative HE and Masson staining images of bone marrow cavities. Black arrow refers to the hematopoietic zone. Yellow arrow refers to the sinusoid. Blue arrow refers to the adipocyte. Green arrow refers to the collagen or reticulin fiber

Next, we deemed to explore the effects and modes of action of SMI, ginsenosides, and carbohydrates on hematopoietic function per se without the interference from tumor signaling. In this regard, 5-FU-induced acute myelosuppressive mouse model was applied (Fig. 2A). 5-FU modeling significantly reduced the BMNC count (*p* < 0.01), while treatment with carbohydrates (S), ginsenosides (Rg) or their combination (S + Rg) significantly increased BMNC count (*p* < 0.05) (Fig. 2B). Figure 2C shows HE staining of the whole femur (1 ×), magnified field of the marrow cavities of both the proximal and distal femurs (40 ×), as well as Masson staining of the distal femur (40 ×). The HE staining revealed that in the Control group, the bone marrow structure appeared normal, all types of hematopoietic cells were evenly distributed in rich hematopoietic zones, and mature erythrocytes were present in the sinusoids. In the Model group, few nucleated cells were in the marrow cavities of both proximal and distal femurs; vascular rupture and serious hemorrhage were observed. The treatment with SMI, S + Rg, S, or Rg resulted in improved structure and morphology of the marrow cavity in the proximal rather than distal femur, along with enhanced angiogenesis, reduced hemorrhage, and increased nucleated cell count. The Masson staining showed that 5-FU modeling led to a large number of reticulin fibers and collagen, which were reduced after SMI, S + Rg, S, or Rg treatment. Notably, the effect on improving bone marrow morphology seemed better when using a combination of carbohydrates and ginsenosides than when using ginsenosides alone. We also noticed that the adipocytes count was greatly increased in the S group compared to that in the Model group (Fig. 2C). Taken together, the results showed that both carbohydrates and ginsenosides in SMI promoted hematopoietic function and improved bone marrow morphology in myelosuppressive mice.

### Carbohydrates promoted the proliferation of BMSCs from acute myelosuppressive mice in vitro

**Fig. 3:**
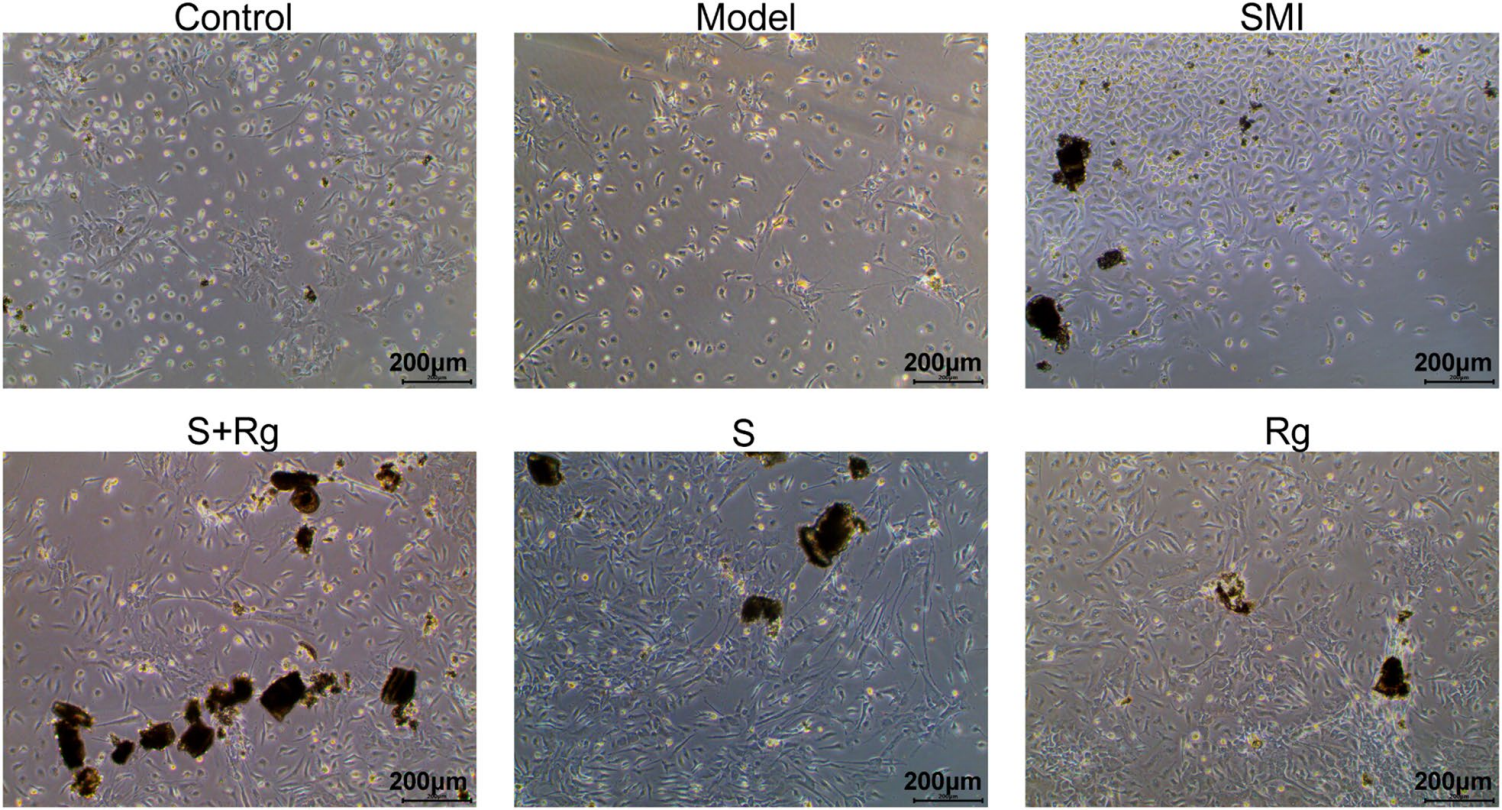
Effects of SMI, ginsenosides, and carbohydrates on the bone marrow cell growth in vitro. Bone marrow cells were extracted from 5-FU-treated mice for long-term bone marrow culture with or without the presence of SMI, S + Rg, S, and Rg respectively. Representative microscopy images of cell growth in each group after 4-week culture are shown. The adhered cells are considered as stromal cells and the resuspended cell clusters are hematopoietic cells

We further investigated the effects of carbohydrates and ginsenosides on the growth pattern of bone marrow cells by long-term bone marrow culture. Figure 3 shows a representative microscopy photograph of the cells in each group: the adhered cells can be recognized as BMSCs and the resuspended cell clusters as hematopoietic cells. Compared to the Control group, the BMSC count in the Model group decreased significantly, and the hematopoietic cell count was very low. Massive proliferation of BMSCs and the formation of hematopoietic cell clusters were observed in the SMI, S + Rg, S, and Rg groups. The result indicated that both ginsenosides and carbohydrates promoted the proliferation of BMSCs and hematopoiesis. Moreover, SMI, and the combination of carbohydrates and ginsenosides appeared to have the greatest effect on promoting the proliferation of bone marrow cells.

### RNA-seq identified transcriptome profiles of carbohydrates and ginsenosides in BMSCs of acute myelosuppressive mice

**Fig. 4:**
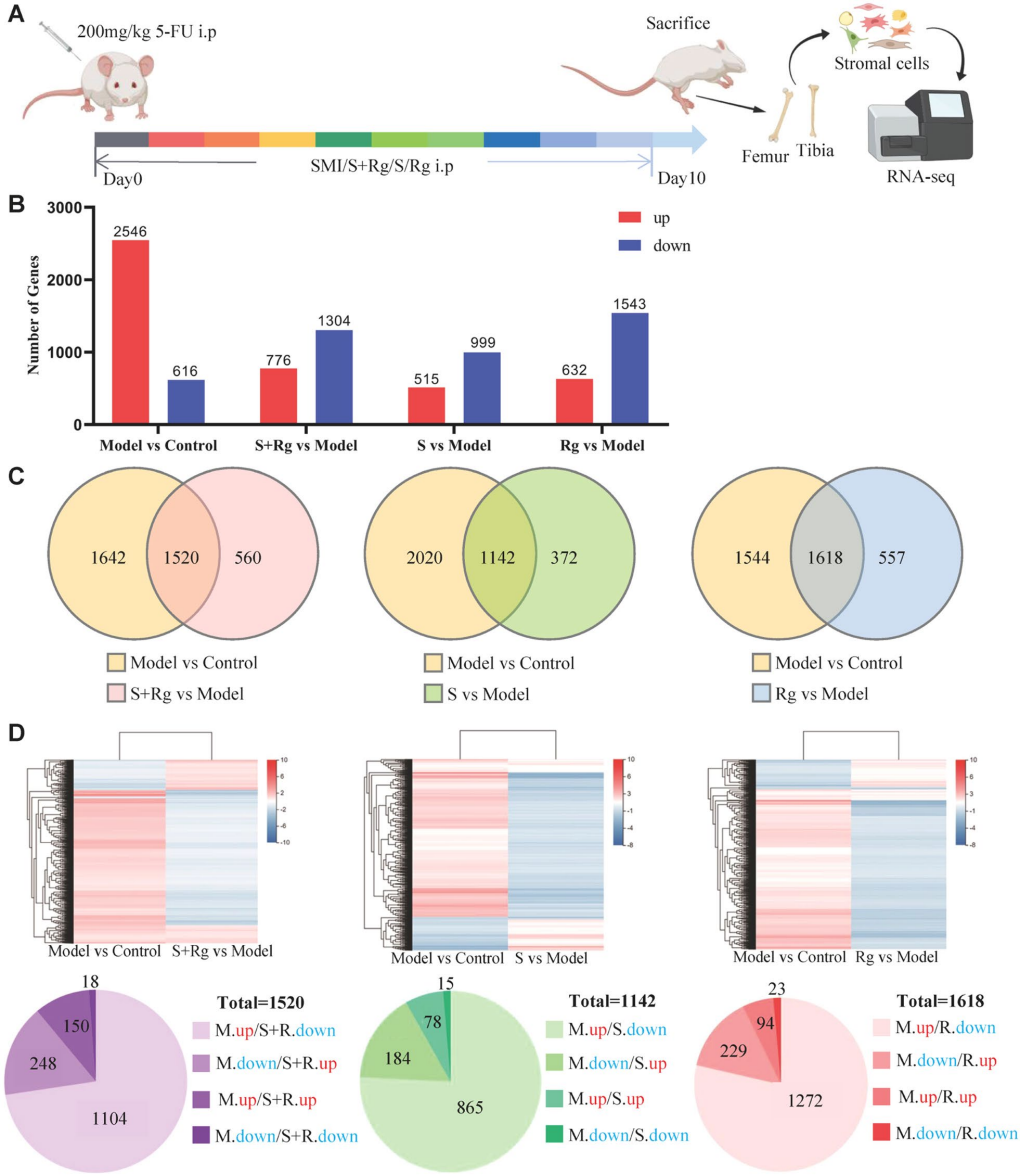
RNA-seq based gene expression profiles of BMSCs obtained from 5-FU-treated mice with myelosuppression. **A** Experimental design. Specific administration is described in “Materials and Methods”. **B** Number of upregulated and downregulated genes in Model vs Control and each treatment group vs Model. **C** Venn diagrams of DEGs between Model vs Control and each treatment group vs Model. **D** Heat maps and Venn diagrams of co-regulated DEGs between Model vs Control and each treatment group vs Model. *M.up* genes are upregulated in Model vs Control, *M.down* genes are downregulated in Model vs Control, *S + Rg.up* genes are upregulated in S + Rg vs Model, *S + Rg.down* genes are downregulated in S + Rg vs Model, *S.up* genes are upregulated in S vs Model, *S.down* genes are downregulated in S vs Model, *R.up* genes are upregulated in Rg vs Model, *R.down* genes are downregulated in Rg vs Model

We used 5-FU to establish an acute myelosuppression model on Day 0 followed by SMI, S + Rg, R, or Rg treatment until Day 10 (Fig. 4A). After confirming the improved hematopoietic function due to the treatment by BMNC count (Additional file 3: Fig. S3), transcriptome profiling analyses of BMSCs were performed to understand the potential mechanisms. We first analyzed DEGs in response to 5-FU-induced myelosuppression with or without treatment. Compared to that in the Control group, 2546 genes were upregulated, and 616 genes were downregulated in the Model group (Fig. 4B and Additional file 5: Table. S2, Model vs Control). Compared to that in the Model group, 776 genes were upregulated, and 1304 genes were downregulated in the S + Rg group; 515 genes were upregulated, and 999 genes were downregulated in the S group; 632 genes were upregulated, and 1543 genes downregulated in the Rg group (Fig. 4B and Additional file 5: Table. S2, S + Rg vs Model, S vs Model, Rg vs Model).

We then analyzed the co-regulated DEGs between each treatment group vs the Model group and the Model group vs the Control group. 1520, 1142, and 1618 DEGs were co-regulated in the S + Rg, S, and Rg groups, respectively (Fig. 4C). Among them, 1104 genes were downregulated, and 248 genes were upregulated in the S + Rg group vs Model group, 865 genes were downregulated, and 184 genes were upregulated in the S group vs Model group, and 1272 genes were downregulated, and 229 genes were upregulated in the Rg group vs Model group (Fig. 4D and Additional file 6: Table. S3). Additionally, as shown in the heatmap in Fig. 4D, the variation trends (upward or downward) appeared to vary similarly between the Model vs Control and Model vs treatment groups. Altogether, the results indicate that ginsenosides, carbohydrates, and their combination can recover the expression of most co-expressed DEGs changed by myelosuppression.

**Fig. 5:**
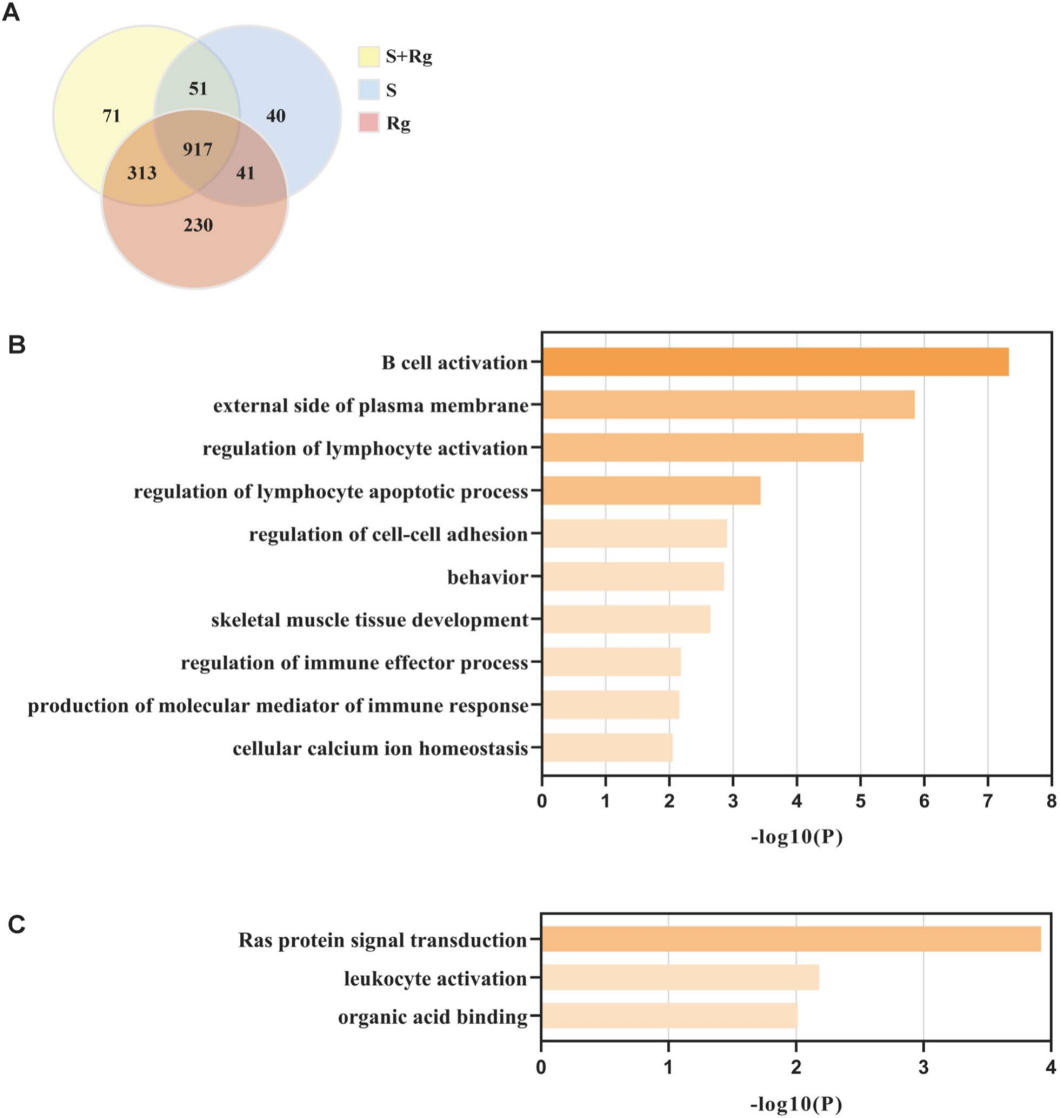
Analysis of DEGs regulated in S and S + Rg groups or uniquely in S + Rg group. **A** Venn diagram of co-regulated callback DEGs between three treatment group and Model group. **B** GO analysis of common DEGs in S and S + Rg groups. **C** GO analysis of DEGs uniquely regulated in S + Rg group

We also analyzed the co-regulated DEGs and their GO functional annotations amongst the treatment groups. As shown in the Venn diagram (Fig. 5A), 51 genes responded to S and S + Rg but not Rg treatment (Additional file 7: Table. S4), and 71 genes responded uniquely to S + Rg treatment (Additional file 8: Table. S5). The GO analysis of the 51 genes indicated 55 GO terms, with the top functional annotation clusters being B cell activation, external side of plasma membrane, regulation of lymphocyte activation, regulation of lymphocyte apoptotic process, regulation of cell–cell adhesion, behavior, skeletal muscle tissue development, regulation of immune effector process, production of molecular mediator of immune response, and cellular calcium ion homeostasis (Fig. 5B and Additional file 9: Table. S6). As for the 71 genes uniquely responding to S + Rg treatment, six GO terms were identified with the top functional annotation clusters being Ras protein signal transduction, leukocyte activation, and organic acid binding (Fig. 5C and Additional file 10: Table. S7). The results support the assumption that carbohydrates may have direct regulatory effect or collaborate with ginsenosides to enable new functions when in combination, and therefore, functionally contribute to the hematopoietic process during chemotherapy-induced myelosuppression.

### Hematopoiesis-related molecular network revealed genes and pathways regulated by carbohydrates and ginsenosides

**Fig. 6:**
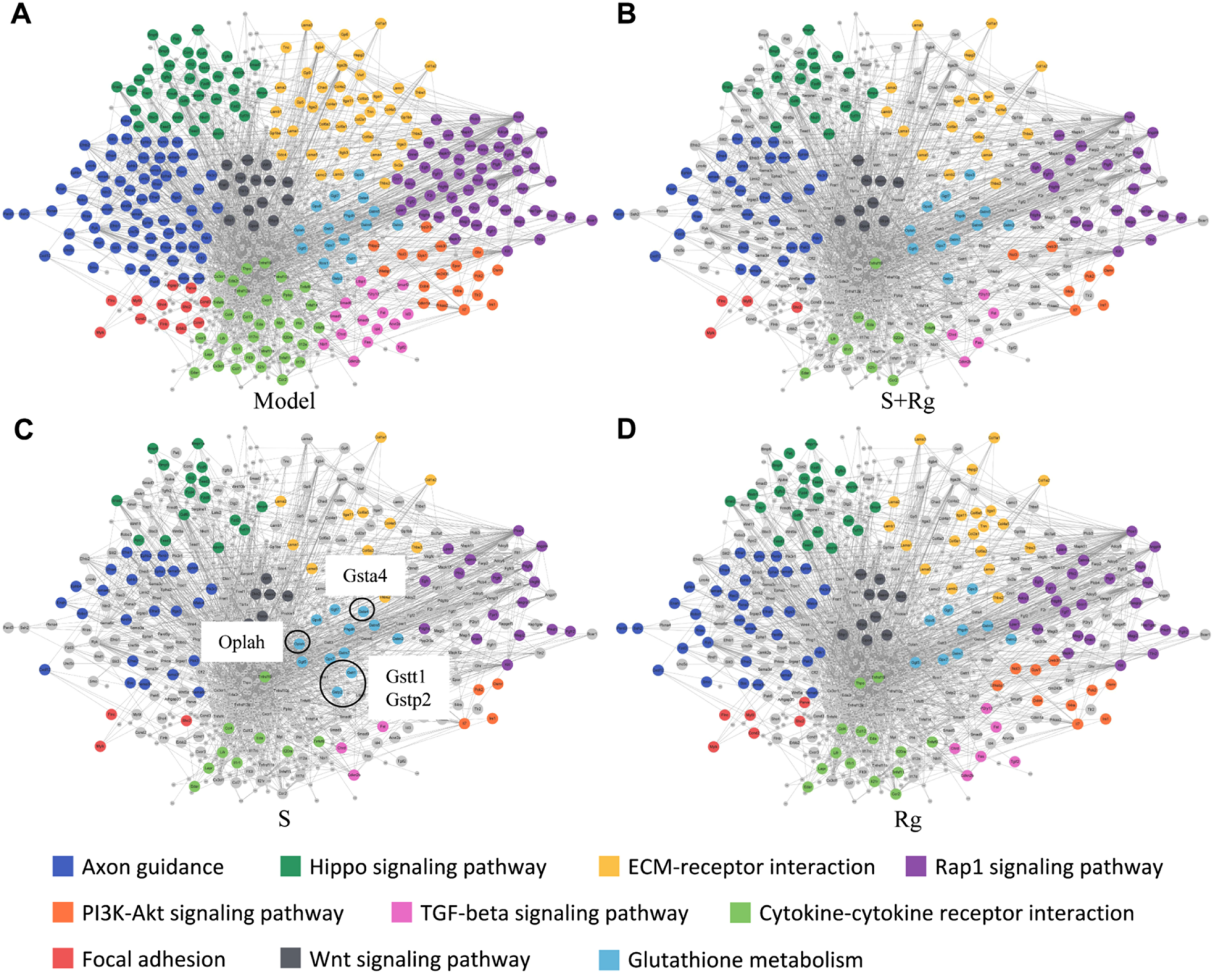
Hematopoiesis-related molecular networks based on genes upregulated in Model vs Control. **A** Model group. **B** S + Rg group. **C** S group. **D** Rg group. Nodes are representative of the genes upregulated in Model vs Control. Lines represent the interaction between the genes. Colors of gene nodes indicate differentially enriched pathways

**Fig. 7:**
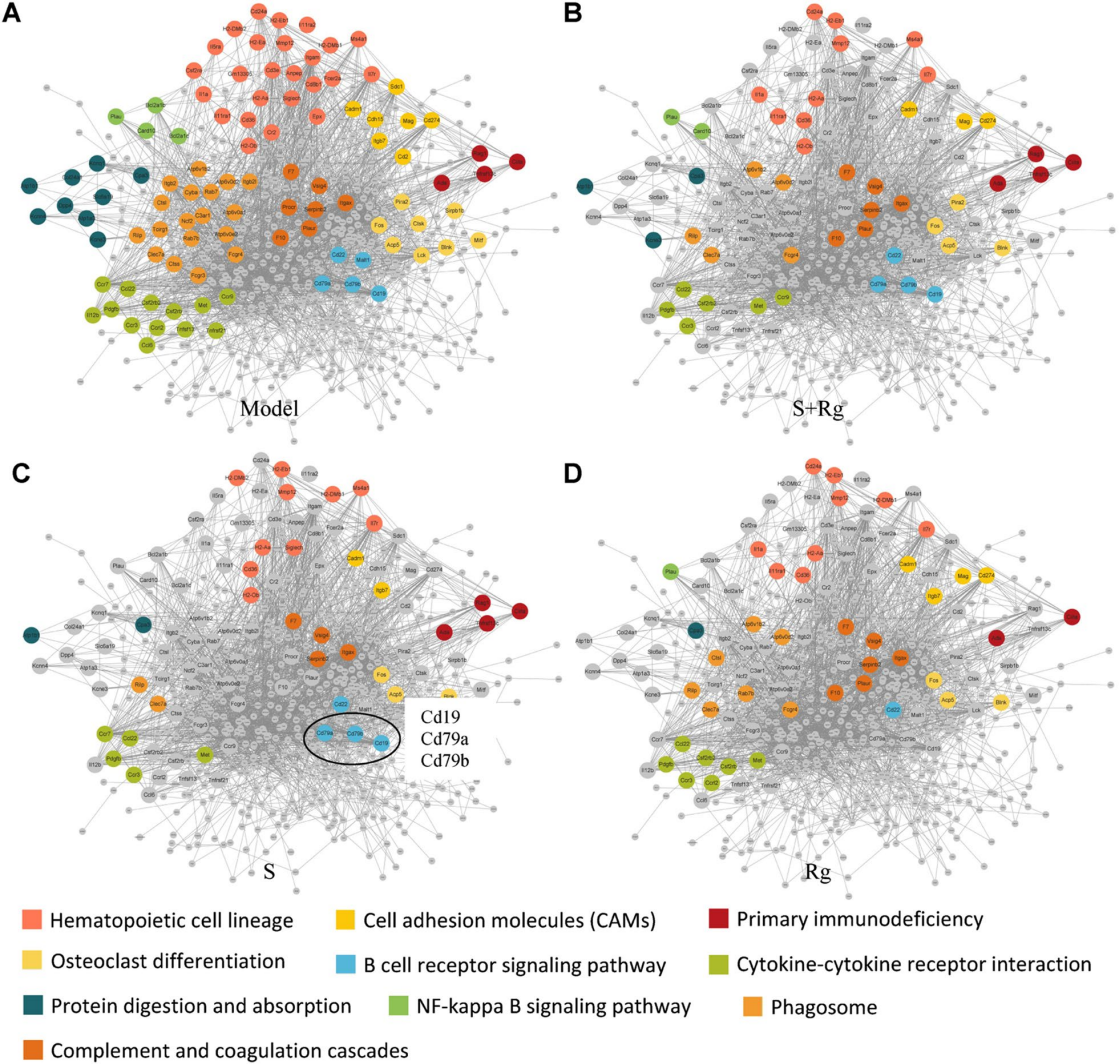
Hematopoiesis-related molecular networks based on genes downregulated in Model vs Control. **A** Model group. **B** S + Rg group. **C** S group. **D** Rg group. Nodes are representative of the genes downregulated in Model vs Control. Lines represent the interaction between the genes. Colors of gene nodes indicate differentially enriched pathways

To further understand if and how carbohydrates contribute to the hematopoietic function of SMI, we built the hematopoiesis-related molecular networks of deregulated genes that were significantly altered in 5-FU-induced myelosuppressive mice. The DEGs caused by myelosuppression were applied to KEGG pathway enrichment (Additional file 11: Table. S8) and interaction network generation (Fig. 6 for up-regulated genes, Model vs Control, Fig. 7 for down-regulated genes, Model vs Control, the main network in gray).

Referring to the enriched activated KEGG pathways, the DEGs related to top ranked pathways, including Axon guidance, Hippo signaling pathway, ECM-receptor interaction, Rap1 signaling pathway, PI3K-Akt pathway, TGF-beta signaling pathway, Cytokine-cytokine receptor interaction, Focal adhesion, Wnt signaling pathway, and Glutathione metabolism were annotated with different colors and highlighted in the network (Fig. 6A, Additional file 11: Table. S8, Model vs Control up). As illustrated in Fig. 6B–D, in which the co-regulated DEGs based on individual treatments are highlighted, the expression of the relevant genes appears to be effectively regulated by individual treatments. We specifically examined the expression patterns of collagen-related genes including *Col1a1*, *Col1a2*, and *Col6a2* in ECM-receptor interaction pathway, as well as genes encoding bone morphogenetic proteins including *Bmp4*, *Bmp5*, and *Bmp6*. These genes were identified as DEGs upregulated due to myelosuppression, which could be regulated by carbohydrates, ginsenosides, and their combination (Additional file 11: Table. S8, S + Rg, S, Rg vs Model down). This result was in accordance with our observation in the histopathological analysis of bone marrow that Masson staining showed that 5-FU modeling led to large numbers of reticulin fibers and collagen, which were reduced after SMI, S + Rg, S, or Rg treatment. We also noticed that amongst the genes in the Glutathione metabolism pathway, *Gstt1*, *Gstp2*, *Gsta4*, and *Oplah* were identified as DEGs regulated by carbohydrates but not ginsenosides (Fig. 6C, D, Additional file 11: Table. S8, S + Rg, S, Rg vs Model down). In accordance with the RNA-seq result, the immunohistochemical staining of bone marrow indicated that the expression of GSTT1 was increased in the Model group compared with the Control group, which could be downregulated by carbohydrates but not ginsenosides (Additional file 12: Fig. S4).

Referring to the enriched inhibitory KEGG pathways, the DEGs related to top ranked pathways, including Hematopoietic cell lineage, Cell adhesion molecules (CAMs), Primary immunodeficiency, Osteoclast differentiation, B cell receptor signaling pathway, Cytokine-cytokine receptor interaction, Protein digestion and absorption, NF-kappa B signaling pathway, Phagosome, and Complement and coagulation cascades were annotated with different colors and highlighted in the network (Fig. 7A, Additional file 11: Table. S8, Model vs Control down). As illustrated in Fig. 7B, D, in which the co-regulated DEGs based on individual treatments are highlighted, the expression of the relevant genes also appears to be effectively regulated by individual treatments. Nevertheless, alternative regulatory effects were observed: NF-kappa B signaling pathway could be regulated by ginsenosides but not carbohydrates; genes involved in B cell receptor signaling pathway including *Cd19*, *Cd79a*, and *Cd79b* could be identified as DEGs regulated by carbohydrates but not ginsenosides. (Fig. 7C, D, Additional file 11: Table. S8, S + Rg, S, Rg vs Model up).

Taken together, these molecular networks demonstrate the effects of carbohydrates, ginsenosides and their combination at the molecular level on hematopoiesis. Carbohydrates in SMI per se may display unique effects in inhibiting the Glutathione metabolism pathway and stimulating the B cell receptor signaling pathway during myelosuppression.

## Discussion

Bone marrow stromal tissue comprises an integral part of the hematopoietic microenvironment that support hematopoietic cell proliferation and differentiation [16]. In addition to the massive reduction in hematopoietic cells, high doses of 5-FU lead to the destruction of bone marrow stromal tissue, the recovery of which can be evaluated based on morphological changes in the bone marrow [17]. In current study, the recovery of bone marrow vasculature structure, the adipogenesis, as well as the alleviated bone marrow fibrosis were selected as signs for morphological changes relevant to hematopoiesis. In detail, the bone marrow vasculature is an important niche for hematopoietic stem cells, and the regeneration of damaged sinusoidal endothelial cells is the rate-limiting step in hematopoiesis after myelosuppression [18]. In addition, adipogenesis is an emergency response that produces niche factors and promotes hematopoiesis in most bones. Compared to constructing new perivascular niches, adipogenesis is a faster way to produce niche factors, which involves the promotion of marrow vascularization [19]. As for bone marrow fibrosis, which is characterized by increased deposition of reticulin fibers, has been reported contributing to the impaired microenvironment favoring malignant hematopoiesis [20]. We revealed that carbohydrates exhibited benefit effect on the recovery of bone marrow stromal tissue morphology, but relatively weaker than ginsenosides. Nevertheless, the effect came out better when using a combination of carbohydrates and ginsenosides than using ginsenosides alone (Fig. 2C). Therefore, carbohydrates collaborate with ginsenosides and jointly contributing to the hematopoietic function of SMI. This assumption was further supported by the finding that Carbohydrates in SMI promoted BMSC proliferation and hematopoiesis in vitro (Fig. 3).

We found that all ginsenosides, carbohydrates and their combination can regulate genes involved in multiple pathways, including ECM-receptor interaction, Hippo signaling pathway, and Wnt signaling pathway (Figs. 6, 7). During hematopoiesis, progenitor cells receive and interpret a diverse array of regulatory signals from their environment. These signals control the maintenance of progenitors and regulate the production of mature blood cells [21]. More than 20 adhesion receptors have been identified on the surface of hematopoietic cells, and their ligands are mainly Extracellular matrix (ECM) and stromal cell surface adhesion molecules [22]. ECM molecules are produced mainly by stromal cells and collagen is the most abundant ECM protein family. The Hippo pathway plays an important role in hematopoietic development, and its activation leads to the inhibition of hematopoietic progenitor differentiation, inhibition of megakaryoblast differentiation, and reduction of platelet biogenesis [23, 24]. The increased expression of some bone morphogenetic proteins is related to Sjögren's syndrome, multiple myeloma, and primary myelofibrosis [25–27]. While the expression of the proteins and their receptors could be detected in both hematopoietic stem cells and stromal cells, Wnt signaling plays a critical role in the differentiation, self-renewal, and maintenance of hematopoietic stem cells [28]. The regulation of those genes and pathways could be recognized as the common mechanism of ginsenosides and carbohydrates activity in SMI against myelosuppression.

*Gstt1*, *Gstp2*, *Gsta4*, and *Oplah* in the Glutathione metabolism pathway could be recognized as genes significantly downregulated by carbohydrates rather than ginsenosides (Fig. 6C, D); the B cell receptor pathway involving *Cd19, Cd79a,* and *Cd79b* appeared to be significantly upregulated by carbohydrates (Fig. 7C, D). Glutathione S-transferases (GSTs) are enzymes involved in detoxification and oxidative stress handling, and their polymorphisms are related to multiple myeloma, aplastic anemia, and acute leukemias [29–31]. Compared with low-growth capacity human mesenchymal stem cells that are *Gstt1*-positive, *Gstt1*-null human mesenchymal stem cells have increased proliferative rates, clonogenic potential, and longer telomeres [32]. As for the B cell receptor pathway, in general, B cells grow and develop in the bone marrow and enter the peripheral circulation after maturation. During development, progenitor cells are localized in the specialized niches of the bone marrow, and process is sensitive to the signals from chemotaxis and/or adhesion to bone marrow matrix molecules [33]. In our current study, it is not surprising that 5-FU injury resulted in oxidative stress and disturbed B cell developmental environment in the bone marrow. Interestingly, both could be rescued by carbohydrates better than ginsenosides, which supports the assumptions that carbohydrates may have a priority in protecting the bone marrow from oxidative stress and stimulating the hematopoiesis of B cells. Nevertheless, both assumptions need experimental verification.

The complex characteristics of Chinese medicine components bring challenges to quality control and safety concerns. Carbohydrates appear to be ubiquitous constituents in TCM injections. In addition to providing energy in the form of glucose, little is known about the biological role of the high amounts of other monosaccharides and disaccharides. To our knowledge, ours is one of the few studies investigated the novel role and mechanism of carbohydrates (fructose, sucrose, and maltose) in promoting hematopoiesis during chemotherapy-induced myelosuppression. Our finding of carbohydrates as bioactive components in SMI helps to decipher the biology of TCM injections and provides a base for further investigations.

Due to the complexity of the intervention setting, we only applied one dosage of SMI by converting from clinical dosage; the mixtures of ginsenosides, carbohydrates, and their combination were prepared at fixed concentrations according to their proportions in SMI. Further investigations are required to verify the bioactivity of carbohydrates, alone or in combination with ginsenosides in tumor-bearing mice. The proposed pathways and genes that contribute to the mode of action of carbohydrates also need to be biologically demonstrated. Despite the study limitations, the present study indicates that carbohydrates may have the ability to improve the bone marrow hematopoietic environment and contribute to the function of SMI. As there might exist other representative active ingredients such as ophiopogonins in Ophiopogon japonicus in SMI, it is interesting to explore the relationship between these constitutes and the action of carbohydrates in the future studies.

## Conclusion

Collectively, we confirmed the hematopoietic effects of SMI on 5-FU-induced myelosuppression in a tumor-bearing mouse model, which supports its clinical application as a supportive medicine for cancer patients undergoing chemotherapy. Importantly, we revealed for the 1st time that carbohydrates (fructose, sucrose, and maltose) may contribute jointly with ginsenosides (Rg1, Re, and Rb1) to the hematopoietic function of SMI. Carbohydrates stimulate BMSC growth, improve bone marrow morphology, and eventually stimulate hematopoiesis during myelosuppression. Carbohydrates may have a priority in inhibiting Glutathione metabolism pathway via *Gstt1, Gstp2, Gsta4* and *Oplah*, and stimulating B cell receptor pathway via *Cd19, Cd79a,* and *Cd79b*. Therefore, carbohydrates could be considered as a bioactive component in TCM injections.

## Supplementary Information

**Additional file 1**: **Figure S1.** HPLC analysis of SMI from different batches. (A) HPLC analysis of SMI (Lot No. 1907018). (B) HPLC analysis of SMI (Lot No. 1907229). (C) HPLC analysis of SMI (Lot No. 1907277).

**Additional file 2**: **Figure S2.** HPLC analysis of carbohydrates in SMI (Lot No. 1907018).

**Additional file 3**: **Figure S3.** BMNC count in each group. Cells were isolated on Day 10 (n = 6 for Control group, n = 3 for Model group, n = 8 for SMI group, n = 6 for S+Rg group, n = 6 for S group and n = 7 for Rg group). The results are expressed as the means ± SEM. ^##^*p* < 0.01, compared with Control group; ^＊^*p* < 0.05, ^＊＊^*p* < 0.01, compared with Model group; ns, no significant difference compared with Model group.

**Additional file 4**: **Table S1.** Substances and their concentrations in SMI (Lot No. 1907018).

**Additional file 5**: **Table S2.** DEGs in Model, S+Rg, S, and Rg groups.

**Additional file 6**: **Table S3.** Co-regulated DEGs among the three-treatment group and Model group.

**Additional file 7**: **Table S4.** Common DEGs in S and S+Rg groups.

**Additional file 8**: **Table S5.** DEGs uniquely regulated in the S+Rg group.

**Additional file 9**: **Table S6.** GO analysis of common DEGs in S and S+Rg groups.

**Additional file 10**: **Table S7.** GO analysis of DEGs uniquely regulated in the S+Rg group.

**Additional file 11**: **Table S8.** KEGG pathway analysis of Model, S+Rg, S, and Rg groups.

**Additional file 12**: **Figure S4. **Representative immunohistochemical staining images for GSTT1 in bone marrows of Control, Model, S+Rg, S, and Rg groups.

## Data Availability

The original RNA-seq data are available in the public database of the NCBI BioProject with project ID PRJNA828339, at http://www.ncbi.nlm.nih.gov/bioproject/PRJNA828339. Other datasets used and/or analysed during the current study are available from the corresponding author on reasonable request.

## Abbreviations

BFU-E: Burst-forming unit-erythroid
BMNCs: Bone marrow nucleated cells
BMSCs: Bone marrow stromal cells
CFU: Colony-forming unit
CFU-GM: Colony-forming unit granulocyte-monocytes
DEGs: Differentially expressed genes
ECM: Extracellular matrix
5-FU: 5-Fluorouracil
GO: Gene Ontology
HE: Hematoxylin–eosin
HPLC: High performance liquid chromatography
KEGG: Kyoto encyclopedia of genes and genomes
RNA-seq: RNA sequencing
SMI: Shenmai injection
TCM: Traditional Chinese medicine
