# Supplementary figures and images for: Carbohydrates and ginsenosides in shenmai injection jointly improve hematopoietic function during chemotherapy-induced myelosuppression in mice
Source: Chin Med. 2022 Nov 4;17:124. doi: 10.1186/s13020-022-00678-5 (PMC9636671; doi:10.1186/s13020-022-00678-5)

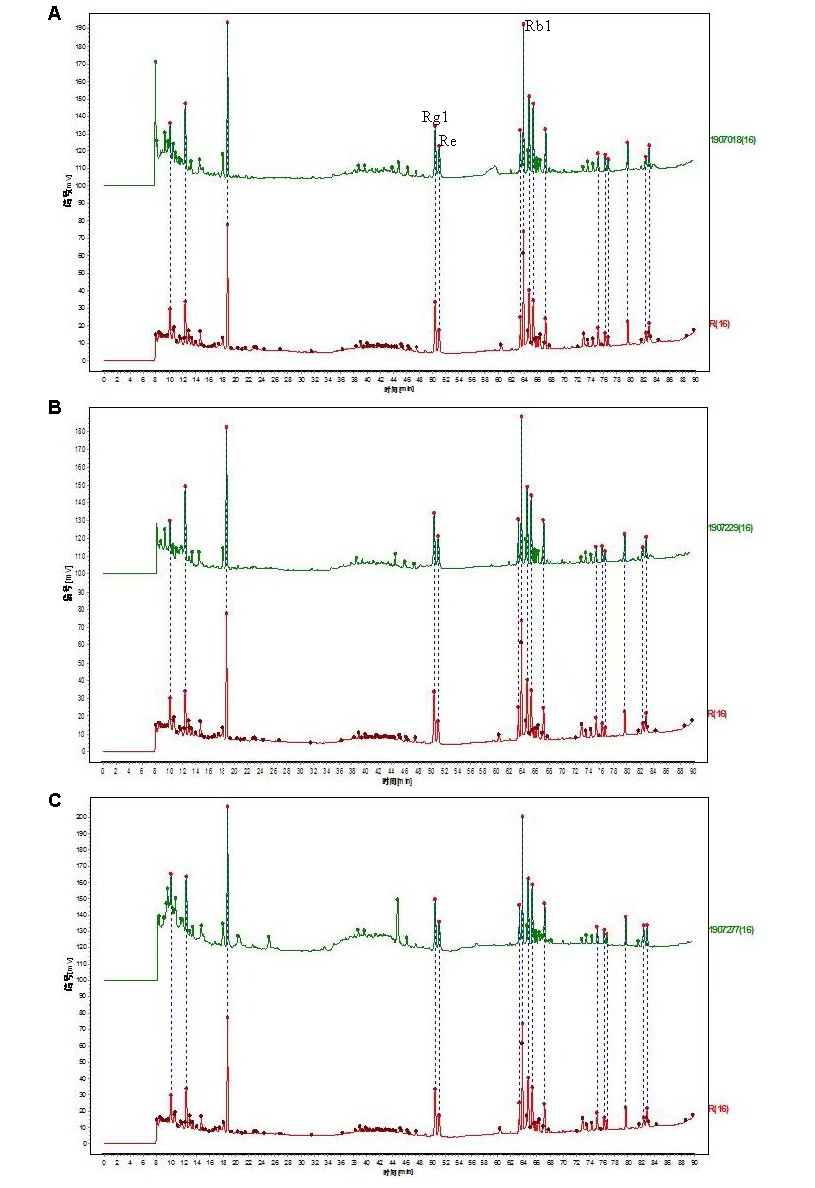

Supplement: Supplementary file 1 — Additional file 1: Figure S1. HPLC analysis of SMI from different batches. (A) HPLC analysis of SMI (Lot No. 1907018). (B) HPLC analysis of SMI (Lot No. 1907229). (C) HPLC analysis of SMI (Lot No. 1907277). [file 13020_2022_678_MOESM1_ESM.tiff]

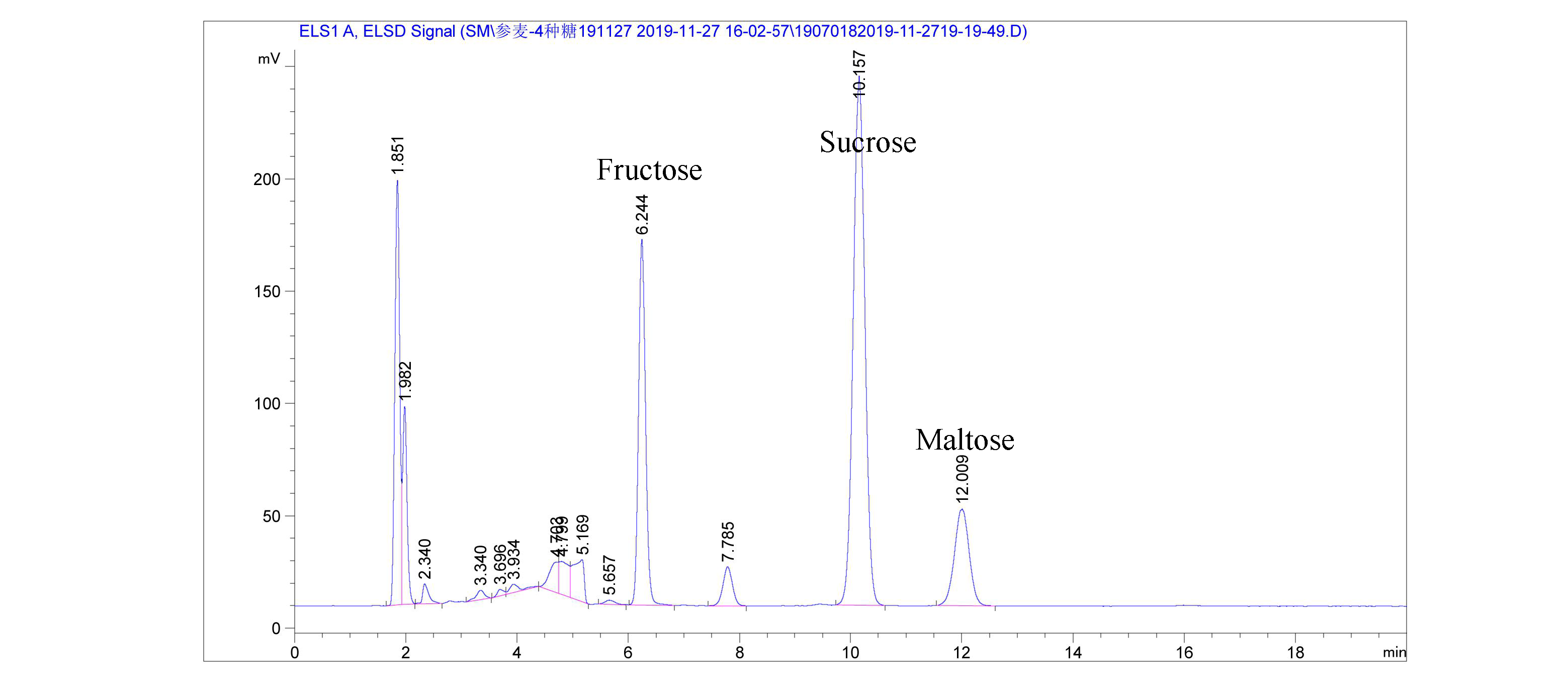

Supplement: Supplementary file 2 — Additional file 2: Figure S2. HPLC analysis of carbohydrates in SMI (Lot No. 1907018). [file 13020_2022_678_MOESM2_ESM.tiff]

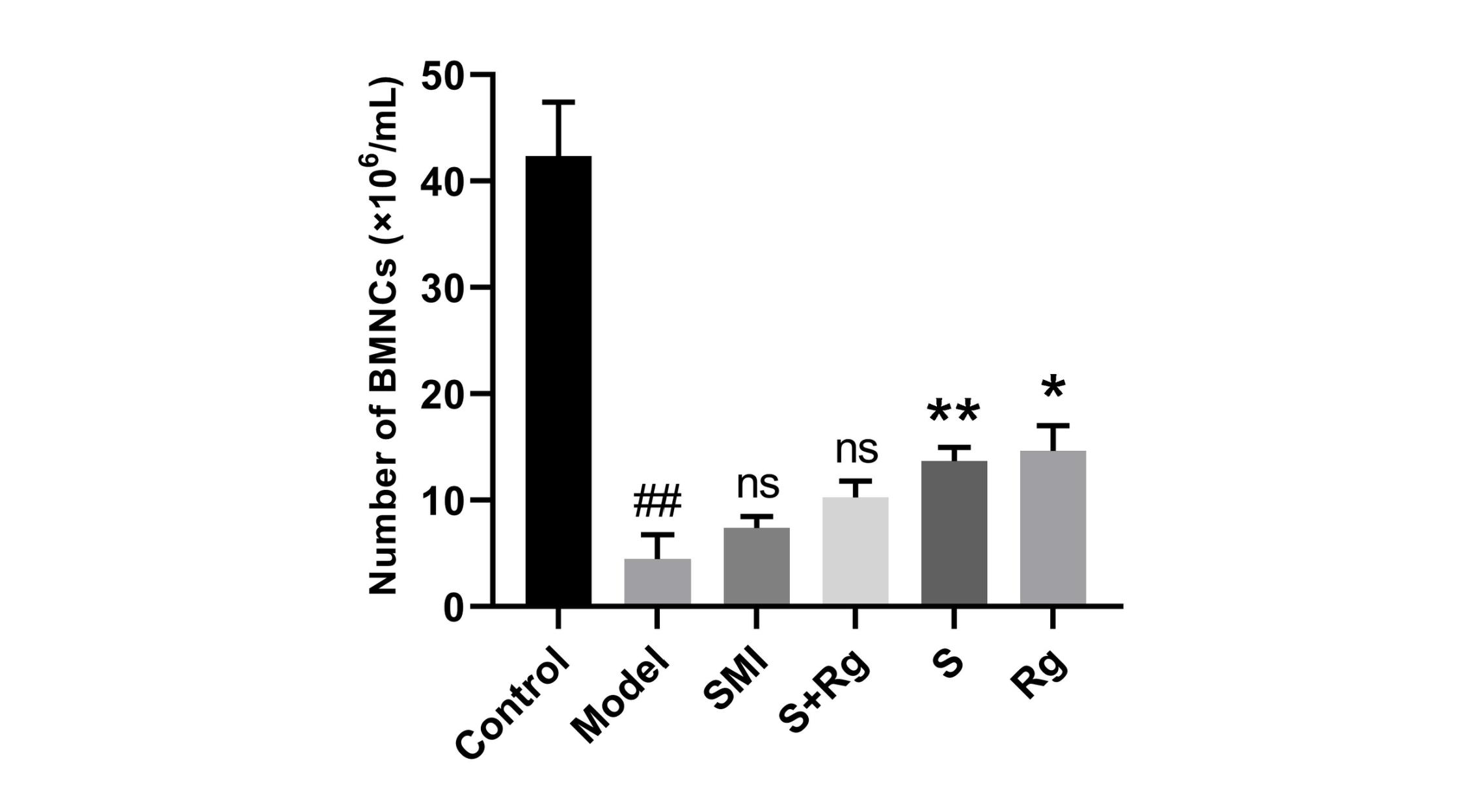

Supplement: Supplementary file 3 — Additional file 3: Figure S3. BMNC count in each group. Cells were isolated on Day 10 (n = 6 for Control group, n = 3 for Model group, n = 8 for SMI group, n = 6 for S+Rg group, n = 6 for S group and n = 7 for Rg group). The results are expressed as the means ± SEM. ##p < 0.01, compared with Control group; ＊p < 0.05, ＊＊p < 0.01, compared with Model group; ns, no significant difference compared with Model group. [file 13020_2022_678_MOESM3_ESM.tiff]

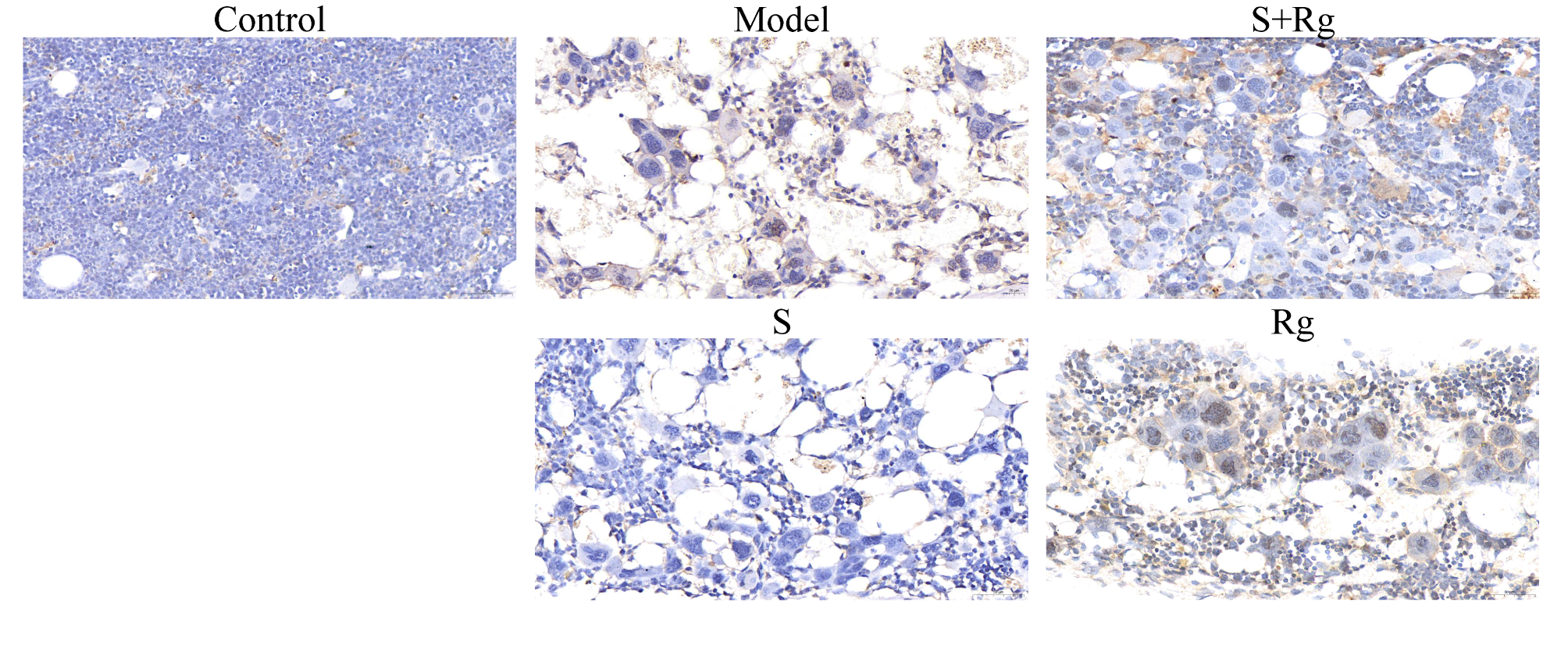

Supplement: Supplementary file 12 — Additional file 12: Figure S4. Representative immunohistochemical staining images for GSTT1 in bone marrows of Control, Model, S+Rg, S, and Rg groups. [file 13020_2022_678_MOESM12_ESM.tif]
