# Supplementary material for: Carbohydrates and ginsenosides in shenmai injection jointly improve hematopoietic function during chemotherapy-induced myelosuppression in mice
Source: Chin Med. 2022 Nov 4;17:124. doi: 10.1186/s13020-022-00678-5 (PMC9636671; doi:10.1186/s13020-022-00678-5)
Supplement: Supplementary file 4 — Additional file 4: Table S1. Substances and their concentrations in SMI (Lot No. 1907018). [file 13020_2022_678_MOESM4_ESM.docx]

Table 1. Substances and their concentrations in SMI (Lot No. 1907018)

|  | Rg1 | Re | Rb1 | fructose | sucrose | maltose |
| --- | --- | --- | --- | --- | --- | --- |
| mg/mL | 0.1594 | 0.1294 | 0.2578 | 3.0169 | 5.9229 | 3.2917 |
| mM | 0.20 | 0.14 | 0.23 | 16.75 | 17.30 | 9.14 |
